# Nanofibrous Biomaterial-Based Passive Cooling Paint Structurally Linked by Alkane-Oleate Interactions

**DOI:** 10.1021/acsami.4c01383

**Published:** 2024-03-01

**Authors:** Andrew Caratenuto, Kyle Leach, Yang Liu, Yi Zheng

**Affiliations:** † Department of Mechanical and Industrial Engineering, Northeastern University, Boston, Massachusetts 02115, United States; ‡ Department of Chemical Engineering, Northeastern University, Boston, Massachusetts 02115, United States

**Keywords:** passive radiative cooling, hydroxyapatite, FTIR, oleate, alkane

## Abstract

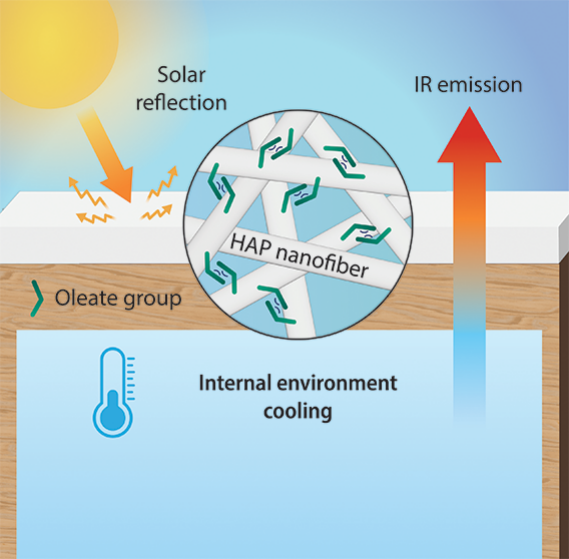

Passive radiative cooling materials, which provide cooling without consuming electricity, are widely recognized as an important technology for reducing greenhouse gas emissions and delivering thermal comfort to less industrialized communities. Optimizing thermal and optical properties is of primary importance for these materials, but for real-world utilization, ease of application and scalability also require significant emphasis. In this work, we embed the biomaterial hydroxyapatite, in the form of nanoscale fibers, within an oil-based medium to achieve passive cooling from an easy-to-apply paint-like solution. The chemical structure and bonding behaviors of this mixture are studied in detail using FTIR, providing transferable conclusions for pigment-like passive cooling solutions. By reflecting 95% of solar energy and emitting 92% of its radiative output through the atmospheric transparency window, this composite material realizes an average subambient cooling performance of 3.7 °C in outdoor conditions under a mean solar irradiance of 800 W m^–2^. The inflammability of the material provides enhanced durability as well as unique opportunities for recycling which promote circular economic practices. Finally, the surface structure can be easily altered to tune bonding behaviors and hydrophobicity, making it an ideal passive cooling coating candidate for outdoor applications.

## Introduction

Reducing human-caused climate change, attributed in large part to substantial greenhouse gas emissions, presents an extremely pressing global issue. A slew of different global consequences are anticipated as a result, with many already beginning to appear, including water insecurity, food production, declining biodiversity, more severe extreme weather events, and detrimental impacts to human health and well-being.^1−6^ Consequently, worldwide efforts to curb emissions and adopt sustainable long-term development strategies are becoming increasingly popular. The energy sector is a particular target for these efforts, as its contribution to climate change and emissions is among the most pervasive^7,8^ – for example, in recent years, electricity generation in the United States has comprised 25% of the country’s emissions, second only to transportation.^9^ Thus, scalable technologies that can reduce electricity usage can have incredibly large impacts on global emission reduction efforts.

A key contributor to global electricity usage is climate control systems for buildings and other structures, such as air conditioning (A/C). Despite the obvious thermal comfort and human health benefits, the technology has comprised about 15% of global electricity use in recent years, making it a massive contributor to greenhouse gas emissions. Moreover, cooling demand is expected to increase over time, due to rising global temperatures, the urban heat island effect, extreme heat events and many other climate change-related factors.^10−12^ Thus, due to widespread and steadily increasing A/C use, sustainable innovations which reduce cooling-related electricity consumption represent a vital step toward mitigating climate change.

Besides the global benefits of reduced electricity use, alternative cooling technologies present further value to communities in hot climates. While 90% of U.S. homes have A/C units, of the population in some of the world’s hottest climates (2.8 billion people), only about 8% have this luxury.^11^ Without sufficient mitigation techniques, high temperatures pose significant health risks to these populations. Annual deaths in excess of 350,000 have been attributed to exposure to excessive temperatures.^13^ Heat-related illnesses and mortality continue to threaten the most vulnerable populations, such as children, the elderly, and underserved or low income communities.^14^ Even in industrialized countries such as the United States, reliance on A/C during heat waves, which may often coincide with blackout conditions, leave many without access to cooling during the most dangerous conditions.^15^ With the expected intensification of climate change, rising global temperatures, and more extreme weather events, such as heat waves, effectively mitigating these heat-related consequences is a paramount humanitarian concern.

Passive radiative cooling (PRC) materials have gained significant academic and industry traction in recent years due to their ability to cool structures without consuming electricity, making them a perfect solution for reducing cooling-related emissions. These materials are engineered with specially tuned spectral properties that allow them to minimize the energy input into structures, as well as maximize their energy output.^16,17^ Some strategies involve specially designed metamaterials to finely adjust the spectral properties. In an early demonstration of subambient cooling, Raman et al. developed a multilayer structure composed of nanoscale layers. By optimizing thicknesses and utilizing several different materials, such as Ag, HfO_2_, and SiO_2_, the material achieved a subambient cooling performance of 4.9 °C.^18^ In another demonstration, Rephaeli and co-workers showed how using photonic structures can help focus infrared emissivity on the atmospheric transparency window, cutting down on parasitic heat input.^19^ On the other hand, the preexisting spectral properties of everyday household materials can also be leveraged for this technique. For example, Styrofoam packaging has recently been repurposed to achieve 96% solar reflectance,^20^ as well as cellulose paper, which exceeded a cooling power of 100 W m^–2^ in an outdoor test.^21^ Efficiency can be further improved by utilizing phase change materials (PCMs), which can adjust spectral properties in response to weather conditions to save energy. Liu et al. showed how the PCM VO_2_ can switch passive cooling properties on and off automatically, depending on ambient conditions.^22^ The same PCM was used by Wang and co-workers within a multilayer structure, who demonstrated the concept for passively switching smart windows.^23^ Also, Tao et al. utilized the PCM dodecanol and a porous nickel foam to form a smart cooling material, which conserves heat in cold environments and rejects heat in hot environments.^24^

For a passive cooling material applied externally on a structure, the net cooling power density can be described as
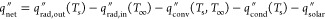
1where *T*_s_ and *T*_∞_ are the surface and ambient temperatures, respectively, *q*_rad,out_^″^(*T*_s_) is the radiative output from the passive cooling material, *q*_rad,in_^″^(*T*_∞_) is the radiative input from atmospheric radiation, *q*_conv_^″^(*T*_s_, *T*_∞_) is the convective input from ambient air, *q*_cond_^″^ is the conductive input from the underlying structure, and *q*_solar_^″^ is the absorbed input from incidence solar irradiance.^25^ Assuming a hemispherical view factor with the ambient environment, the radiative and solar components can be expanded as^16,25^
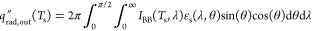
2
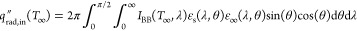
3
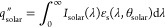
4
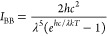
5where *I*_BB_ is the blackbody radiation from Planck’s Law, λ is the wavelength, θ is the incident angle of radiation, ε_*s*_ and ε_∞_ are the emissivities of the passive cooling surface and atmosphere, respectively, and *I*_solar_ is the incident solar irradiance. Following from Kirchhoff’s Law, the emissivity ε is assumed to be equal to the absorptivity α.^25^

Thus, the resulting cooling power is heavily dependent upon weather conditions (especially the solar irradiance and ambient temperature) as well as the material properties of the passive cooling material (especially the solar irradiance and infrared emittance). Clearly, maximizing the reflectance in the solar wavelength region severely reduces the energy input, which is desirable. For reasonable terrestrial passive cooling surface temperatures, Planck’s Law indicates that most power will be radiated at the lower end of the mid-IR range. Therefore, while a high emittance in the infrared region is desirable to maximize energy output, atmospheric radiation provides another potential source of energy input in a similar wavelength region, which should ideally be avoided. The Earth’s atmosphere exhibits very high transparency in the region of 8–13 μm.^17^ Hence, effective passive cooling materials will take advantage of this region of low incident atmospheric radiation, aiming to maximize their emittance in this wavelength region while simultaneously minimizing their emittance in other regions to avoid absorbing incident radiation from the sun and the ambient environment.

If all parasitic thermal loads are eliminated, this technique has the theoretical capability to realize subambient temperature reductions up to 60 °C.^26^ However, practical material applications are generally limited temperature drops lower than 10 °C, primarily due to heat input from convection, conduction, and nonideal spectral properties.^16^ A great variety of passive cooling materials have been proposed in recent years, including finely tuned nanostructures and metamaterials,^27,28^ ultrareflective paints,^29^ and naturally derived materials.^21^ However, for widespread practical applications, passive cooling performance must be balanced with factors including cost, material sustainability, scalability, and durability. With this in mind, the biomaterial hydroxyapatite is a key candidate for practical passive radiative cooling. Besides its desirable spectral properties, its biomaterial properties and high temperature resistance afford this material significant value as a passive cooling material.^30,31^

Several recent studies have exemplified the desirable properties of hydroxyapatite (HAP) in passive cooling applications. Sun et al. demonstrated a cellulose-based paper with embedded nanoscale HAP, achieving a solar reflectance and infrared emittance of 0.94 and 0.95, respectively.^32^ Tang et al. illustrated how a hydroxyapatite could be colored using other pigment materials, while still preserving a relatively high reflectivity.^33^ Most relevant to this work, a nanofibrous form of hydroxyapatite achieved a normalized solar reflectance of 0.99, offering near-ideal passive cooling properties and allowing the material to achieve a subambient temperature drop of 5.1 °C outdoors.^31^

While the spectral properties of nanofibrous hydroxyapatite are close to ideal for passive cooling, the material itself must be combined with a delivery method that offers ease of application in practical scenarios. A simple, scalable application method will help guarantee global adoption, which is vital for realizing the true environmental and humanitarian benefits of passive radiative cooling. One of the easiest and most universal methods for applying exterior-facing materials at scale is offered by paints and coatings. In general, paints consist of a binder, which forms a film to hold the components of the paint together, pigments, which provide color and opacity, and solvents, which help to control viscosity and guarantee ease of application. Additives may also be included to further modify paint properties, such as defoamers and drying agents. Binders are either water-based (usually acrylic), which mainly use water as a solvent, or oil-based, which tend to use organic compounds as solvents, such as turpentine.^34^ Linseed, safflower, poppy, and walnut oils are typical oil paint bases, especially for applications in art.^35,36^ Acrylic paints are generally more common for consumer applications.^37^

**Figure 1:**
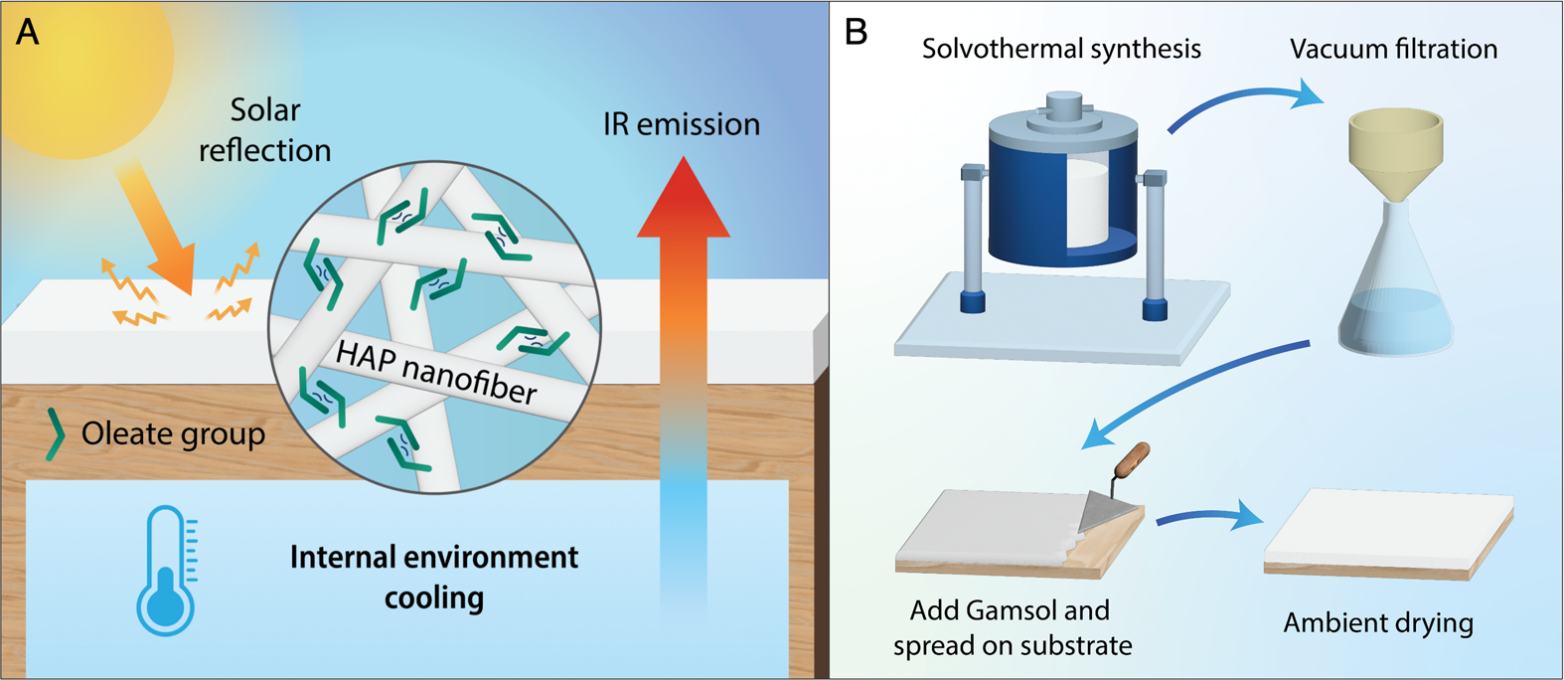
HAP nanofiber paint for passive cooling. (A) Schematic representation of HAP/Gamsol paint applied in an external environment. (B) Fabrication process of the HAP/Gamsol paint.

A key challenge in composing paints that achieve passive radiative cooling relates to the binder. Many pigments with near-ideal solar reflectance and infrared emittance exist, but most binders will introduce unavoidable optical absorption (especially in the near-IR region).^38^ A key challenge is therefore to develop a paint that incorporates high-performance PRC pigments while minimizing the optical influence of the binder. This is further complicated by the relatively low refractive index of many investigated PRC pigments, as well as the practical limitations on pigment volume fraction in binder media.^38,39^ In this work, the implementation of nanofibrous HAP as a pigment material within various paint matrices is studied to establish a viable delivery method for passive cooling solutions. The impact of several types of paint bases is studied with respect to relevant spectral properties. Mechanisms of paint media bonding with HAP are studied in great detail using FTIR and other characterization methods. It is discovered that adsorbed surface compounds on the HAP provide it with unique bonding attributes not shared by many other pigment materials. Bonding mechanisms for HAP-based paints are proposed, and recyclability and surface property modification of HAP are illustrated. Through this study, a mechanically stable HAP-based paint which maintains 95% solar reflectance and 92% infrared emittance in the atmospheric transparency region is developed (Figure 1a). Finally, the cooling capabilities of this composite coating are demonstrated in both indoor and outdoor environments to clearly exemplify the sustainable benefits of this unique HAP-based paint.

## Results

### Material Properties

**Figure 2:**
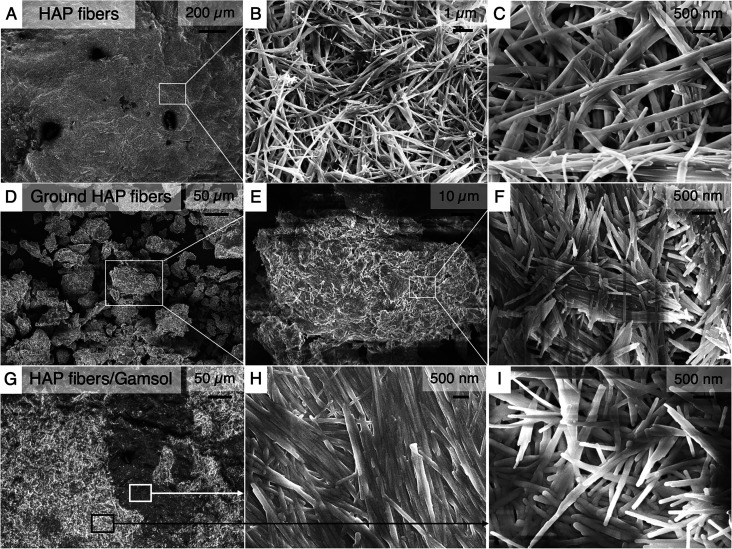
SEM images. SEM images of (A–C) as-synthesized HAP fibers, (D–F) HAP fibers after grinding with a mortar and pestle, and (G–I) after combining with Gamsol and drying.

The aforementioned fabrication procedure yields HAP fibers with high aspect ratios, consistent with similar procedures in the literature.^40−43^ The morphology of the as-synthesized fibers is shown in the SEM images (Figure 2a–c). Fiber diameters are generally below 100 nm, while their lengths are in the low micron range. The Mie theory can be used to predict fundamental scattering parameters of materials of various scales and geometries, provided that the relevant length scales are comparable to incident wavelengths. As such, the Mie theory is employed often to evaluate micro and nanoscale features in response to solar wavelengths.^34^ Previous investigations of the same fibrous HAP used in this work have illustrated the benefits of the fibrous morphology oversimple spherical particles.^31^ The main advantage of this morphology is the introduction of enhanced broadband scattering performance compared to single particle sizes. For HAP, feature sizes around 50 nm provide strong reflectance for smaller solar wavelengths (UV and low visible regions), while feature sizes around 1 μm are well suited to scatter longer visible and near-IR wavelengths. The fibers produced in this work possess both length scales, providing a strong broadband scattering performance over the entirety of the solar wavelength spectrum. In addition, the tendency of fibers to overlap provides enhanced fiber–fiber interfacial area, offering added paint strength due to larger bonding areas and the potential for interweaving interactions. This characteristic also introduces a very high density of air voids within the structure at fiber-air interfaces, which have been illustrated to further enhance photon backscattering.^31^ Thus, the fibrous morphology provides both broadband solar reflectance and enhanced structural stability, both of which strengthen its application in PRC paints.

Once the HAP fibers are ground using a mortar and pestle, the large bulk surface transforms into a particle distribution, with sizes from about 5–50 μm (Figure 2d). While this practice results in fibers clumping together, the fibrous morphology is still preserved on the nanoscale (Figure 2e, f). Once the Gamsol is added, and the mixture is spread onto a substrate, the particles appear to merge together quite successfully into a continuous bulk surface (Figure 2g–i). Two distinct regions can be seen at low magnification in the HAP/Gamsol mixture: one shows a more close-packed, ordered distribution of fibers (Figure 2h), while the other shows a more randomly oriented fiber distribution with more gaps (Figure 2i). The relative randomness of these regions is likely due to the hand-mixing process of the HAP and Gamsol using a trowel. While the close-packed region may provide additional structural stability for this paint mixture, the randomly oriented regions with air gaps likely contribute to a higher scattering efficiency.^31^

**Figure 3:**
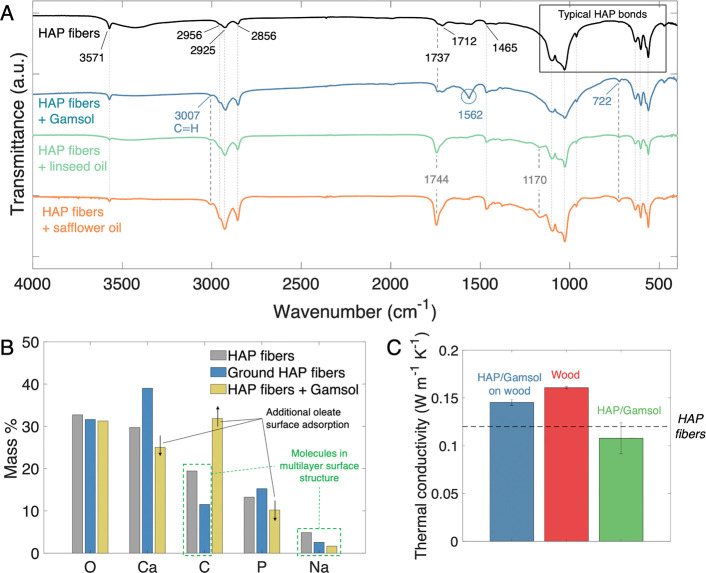
Material characterizations. (A) FTIR transmittance, (B) EDS, and (C) thermal conductivity of various HAP and HAP paint samples.

To investigate the material properties and chemical bonding behaviors of the prepared HAP paint, Fourier-transform infrared spectroscopy (FTIR) and energy-dispersive X-ray spectroscopy (EDS) are performed (Figure 3). FTIR transmittance measurements are obtained using a potassium bromide (KBr) window. Both the HAP fibers and various HAP/paint formulations show absorption peaks corresponding to the typical bonds of HAP. The signature at 3571 cm^–1^ corresponds to the hydroxyl group of HAP, while the peaks at 1097, 1028, and 962 cm^–1^ are ascribed to symmetric and asymmetric stretching modes of the phosphate group. The phosphate group also shows a bending mode, which is seen via the absorption peaks at 634, 603, and 561 cm^–1^.^41,44,45^ These observations are in agreement with EDS results, which indicate large quantities of calcium, oxygen, and phosphate, as is expected for HAP.

In addition to the FTIR signatures expected from HAP, several other notable peaks are observed in all samples. Absorption peaks at 2956, 2925, 2856, 1737, 1744, 1712, and 1465 cm^–1^ all indicate the presence of carbon within the structure, which is corroborated by the EDS results. As a result of the oleic acid present during the reaction, carbon-containing oleate compounds are often adsorbed onto the surface of HAP structures synthesized using similar solvothermal procedures.^40−42^ This adsorption mechanism involves the bonding of calcium ions on the surface of the apatite with oleate ions in the solution.^42,46−48^ In such cases, the most commonly observed peak locations are 2925 and 2856 cm^–1^, which are specifically attributed to CH_2_ stretching vibrations.^40−43,49−51^ These resonances typically arise from calcium oleate in these types of reaction systems. Based upon the sodium detected in the EDS measurements, it is likely that sodium ions are also incorporated into the oleate surface structures, whose charges are balanced by phosphate ions.^48^

Notably, the HAP nanofibers synthesized here show additional peaks at 2956, 1737, and 1712 cm^–1^. The weak CH_3_ peak at 2956 cm^–1^ may manifest from either oleic acid or oleate compounds. In either case, its visibility is indicative of the high quantity of carbon compounds on the apatite surface. The strong pair of peaks at 1737 and 1712 cm^–1^ indicates oleic acid itself, rather than surface-adsorbed oleates, as these are resonances of the carboxyl group found in oleic acid.^37,42,47,52−54^ When oleates form on the surface of apatites, oleic acid monomers and dimers may adsorb onto the surface of these oleates. Rinsing the HAP after vacuum filtration may remove the acid, explaining why these peaks are not typically noted when comparable synthesis methods are employed.^47,54^ In this work, the rinsing step was omitted to maximize carbon-based compounds on the HAP surface, as their presence was found to be critical to forming a solidified paint.

The EDS results also corroborate these surface-adsorption hypotheses. When the HAP fibers are ground from the bulk form to micrometer-sized particles, their surface structures will be disturbed, and fibers may be partially crushed or cleaved, opening their inner surfaces. As a result, both calcium and phosphorus, the pure HAP constituents, increase in mass after grinding, whereas carbon and sodium both decrease in quantity. The oxygen content is only slightly modified, as oleates, oleic acid, and pure HAP all contain oxygen. This helps to confirm that carbon-based compounds are present on the surface of the HAP fibers as opposed to within their fibrous structure.

To bind the HAP fibers together and produce a paint-like substance, Gamsol (odorless mineral spirits), linseed oil, and safflower oil are used. Gamsol is composed of alkanes obtained through petroleum distillation, made up of various saturated hydrocarbons (mainly C11–C13), in which nearly all aromatic compounds are removed. Linseed and safflower oils are drying oils composed of various fatty acid compounds. Both consist primarily of unsaturated fatty acids (mainly linoleic and oleic), which account for 80–90% of the composition.^55,56^ When oil paint bases are mixed with pigments, autoxidative processes will occur, drying the mixture over time. As the mixture begins to dry, hydrogen atoms abstract from the fatty acids, forming free radicals within the structure. Ambient oxygen will then bond with the newly formed free radicals to form peroxides and cross-link the structure.^35,37,57^ When the drying process is complete, a hardened surface binding the pigments together will result. Notably, Gamsol is typically used only as a thinner for other paint bases and not as a standalone binder. As will be shown in the following sections, unique interactions with HAP and its surface compounds allow Gamsol to facilitate the formation of a HAP-based coating through a different mechanism than those of typical oil paints.

Certain spectral features of the various HAP-based paints are in agreement: namely, new peaks around 722 and 3007 cm^–1^, as well as a notable enhancement of the peak at 1465 cm^–1^. The activity at 722 and 1465 cm^–1^ indicates CH_2_ vibrations, brought on by additional methylene groups from the added hydrocarbon compounds,^37,48^ while the new peak at 3007 cm^–1^ is a C–H olefinic stretch. The latter is a common marker of peroxidation processes in oil paint drying, but more generally corresponds to the C=C double bond of an unsaturated hydrocarbon.^36,47−49^ One key difference between the Gamsol-based and oil-based paints is the carboxylic acid peak at 1170 cm^–1^, indicative of the fatty acids of the oil bases which are not present in alkanes such as Gamsol.^45^ In addition, there are important differences around 1737–1744 cm^–1^. While the Gamsol paint retains the original 1737 cm^–1^ peak, indicating oleic acid on the surface, the oil-based paints show a shift to 1744 cm^–1^. This latter activity is specifically associated with the peroxidation of fatty acid chains during paint drying processes,^49^ explaining why the oil-based paints (composed of fatty acids) show this peak while the Gamsol-based paint (composed of saturated hydrocarbons) does not. The lack of resonance at 1744 cm^–1^ in the Gamsol paint, as well as the weak shoulder near 1737 cm^–1^ in the oil-based paints (Figure S1) also suggest that the small quantity of oleic acid contained on the surface does not undergo peroxidation processes that are significant to the structure of the paint.

While the drying and bonding mechanism of the oil-based paints with the HAP fibers agree with standard processes for oil paint drying, the question of the bonding mechanism of the Gamsol-based paint remains. It is clear that HAP/Gamsol bonding results in the addition of functional groups which exhibit C–H stretching and CH_2_ vibrations and contain C=C double bonds, and is unlikely to involve fatty acid chain peroxidation. Additionally, a very prominent peak at 1562 cm^–1^ is unique only to the HAP/Gamsol paint, which indicates the presence of carboxylate groups.^53,58,59^ The emergence of this peak coincides with a reduction in the broad O–H stretching vibration of adsorbed water, which is commonly present on the surface of HAP through both hydrogen bonding with hydroxyl groups and ion-dipole interactions with Ca and phosphate groups.^60,61^ The 1562 cm^–1^ peak is therefore attributed to further oleate formation on the surface of the hydroxyapatite. While the carboxyl groups of calcium oleate often show a dual peak in this region, this strong single peak is specifically associated with surface oleates forming under conditions of low water content, as well as sodium oleate.^47,48,54^

Based upon these data, the hypothesized bonding mechanism for the HAP/Gamsol paint is as follows. The morphology of the HAP surface structure after vacuum filtration is likely amorphous, randomly oriented, and contains a variety molecules (oleic acid, sodium and/or calcium oleate, water, and phosphate, calcium, and sodium ions).^48^ The addition of Gamsol, an alkane with hydrophobic/nonpolar properties, may disrupt this surface structure, breaking apart aggregate molecules, forming weak bonds with other hydrocarbon chains, and temporarily displacing water molecules, as will be shown in later sections. In addition, the hydrophobic nature of both alkanes and the nonpolar tail of oleate compounds provides opportunities for weak interactions between these groups. The introduction and subsequent autoxidation of this alkane evidently facilitate the adsorption of further oleates on the surface of HAP, evidenced by the strong resonance at 1562 cm^–1^. This may be enabled simply by the disruption and reorganization of the surface structure, which would allow oleates suspended within the surface structure the opportunity to bond directly with the HAP surface. The surface structure likely remains amorphous, indicated by its hydrophilicity (Figure S2), relatively high peak locations of 2925/2853 cm^–1^, and low water content.^48,52^ The olefinic C–H and CH_2_ markers that emerge (3007 and 722 cm^–1^, respectively) are most likely attributed to the additional surface oleates. Thus, the weak molecular interactions that may result between newly formed oleates in the presence of Gamsol are the most likely causes of the hardened paint surface after drying. The hydrocarbon tails of oleates may interact with one another and form oleate dimers, van der Waals bonds, or tail–tail hydrocarbon bonds, which could be facilitated by nonpolar alkanes prior to their evaporation.^62−64^ The fact that water content remains low is one marker of further oleate adsorption on apatite surfaces, which provides further evidence for the alkane facilitation of new surface-adsorbed oleate compounds.^48,54^ However, it is further hypothesized that the proposed intermolecular oleate interactions play a role in minimizing HAP water adsorption. Thus, we posit that, as opposed to the cross-linking processes of the linseed and safflower oil-based paints, the Gamsol introduces a stable coating surface by facilitating interfiber oleate interactions, allowing the coating to be held together via weak molecular interactions. Further discussion of these mechanisms is provided in later sections.

### Thermal and Spectral Performance

A relatively high thermal conductivity is often beneficial to passive cooling materials as it facilitates thermal energy transport from indoor areas to the outside. The previous work studying the passive cooling performance of bulk HAP fibers notes the benefits of its relatively high thermal conductivity.^31^ Adding Gamsol to create a paint formulation has little impact on the thermal conductivity (Figure 3c). It is difficult to remove a large, continuous layer of HAP/Gamsol paint from the substrate after drying for subsequent characterization of the thermal conductivity. For this reason, the thermal conductivity of the paint is first tested on the wood substrate, followed by the wood substrate alone. By measuring the thickness of each layer and assuming the thermal resistance of the composite acts in series, the thermal conductivity of the HAP/Gamsol paint alone can be obtained.^65^ It is found that the thermal conductivity of the paint is largely unchanged with respect to the bulk HAP fibers, as the values agree within about 0.02 W m^–2^ K^–1^.^31^

**Figure 4:**
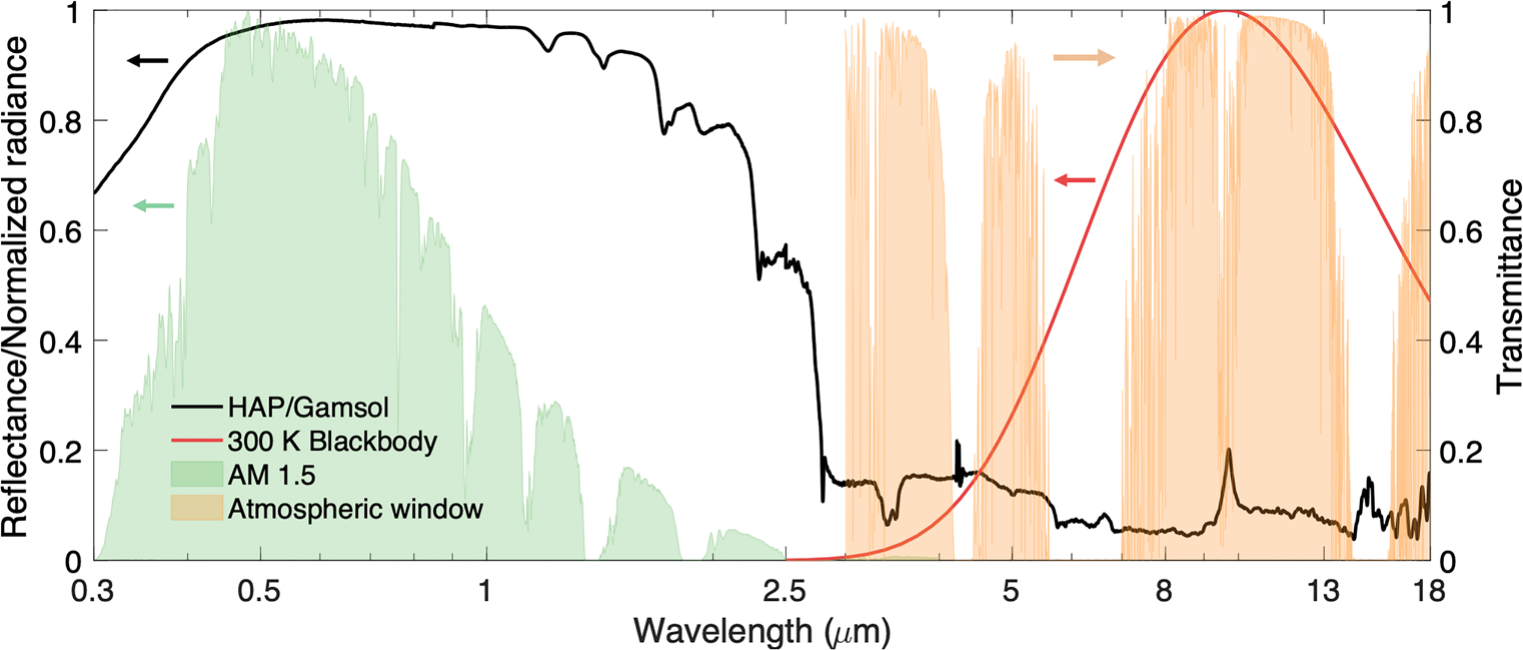
Spectral performance of HAP paint. Reflectivity of HAP/Gamsol paint, shown with respect to a 300 K blackbody, normalized AM 1.5 solar radiance, and the normalized atmospheric transparency window.

The spectral performance of the finalized HAP/Gamsol paint is shown in Figure 4. This paint formulation has superb solar reflectance in the entire AM 1.5 solar spectrum, providing a normalized reflectivity value of 95%. A high infrared emittance is also obtained, largely via the contributions of infrared vibrations from both phosphate compounds and carbon-based surface structures. The high emittance agrees well with the atmospheric window (approximately 8–13 μm) as well as the emission spectrum of a 300 K blackbody, providing strong heat rejection performance in the most relevant infrared region for terrestrial cooling.

**Figure 5:**
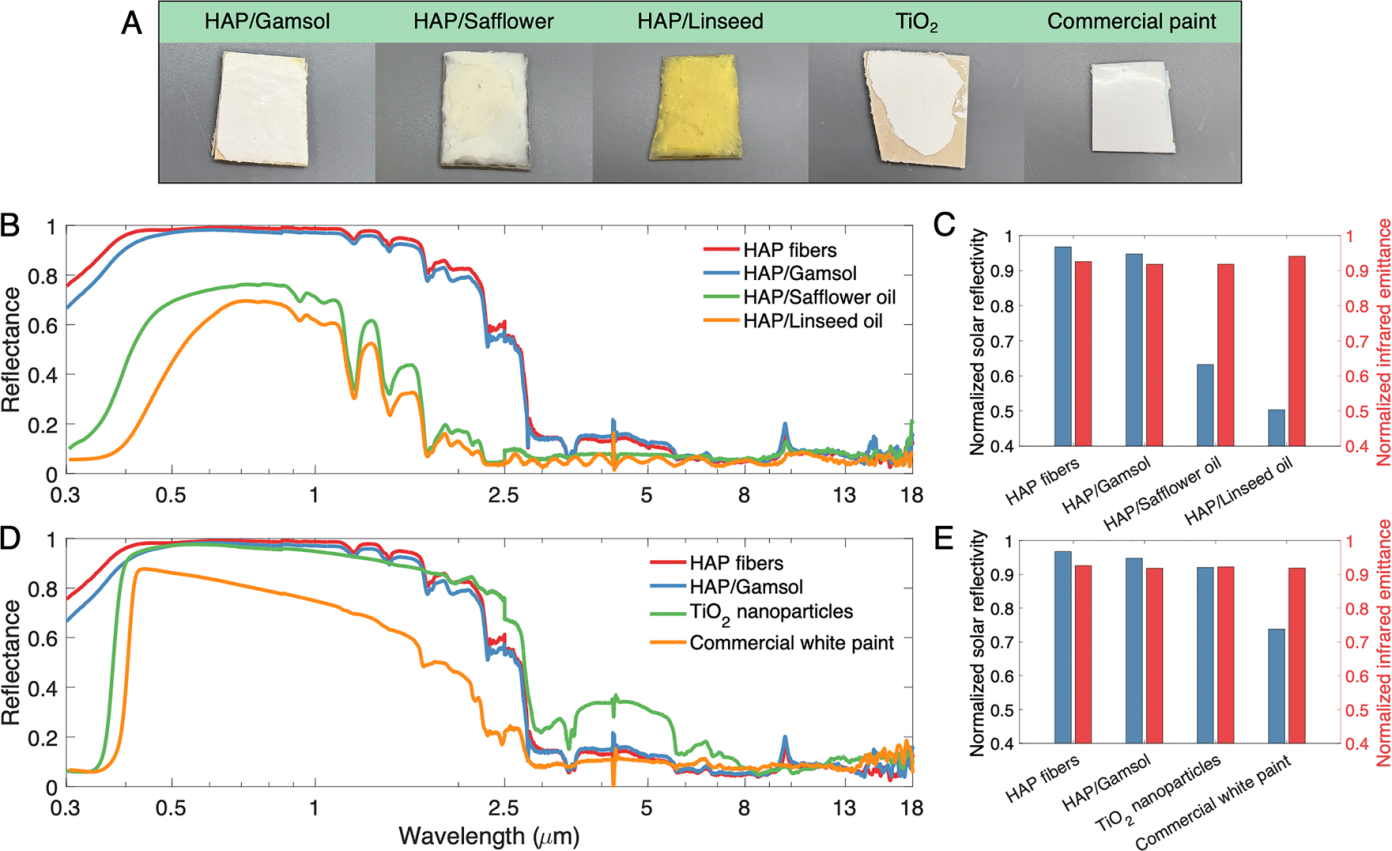
Spectral performance of all paint samples. (A) Images of various paint and comparison samples. (B) Spectral and (C) normalized reflectance and emittance of HAP fibers and paints and (D) spectral and (E) normalized reflectance and emittance of HAP fibers and Gamsol paint in comparison to industry comparison samples.

In comparison to other paint formulations (Figure 5), HAP/Gamsol exhibits the strongest passive radiative cooling properties. Its IR emittance is virtually unchanged from synthesized bulk fibers, and its normalized reflectivity drops by less than 2%—most significantly at wavelengths of higher energy. In contrast, both the safflower and linseed oil formulations perform far worse than the bulk fibers. This is obvious via visual inspection (Figure 5a)—the HAP/safflower paint becomes quite dull after drying, and the HAP/linseed paint yellows significantly. This manifests clearly in the measurements, as these mixtures yield normalized reflectivity values under 70% and 60%, respectively. While their IR emissive properties are slightly more favorable than the HAP/Gamsol paint, such a large increase in absorbed solar irradiance would render them ineffective passive cooling mixtures.

The HAP/Gamsol mixture is also compared with two industry benchmarks: titanium dioxide (TiO_2_), a widely used white pigment, and a standard semigloss white paint. The commercial white paint, though visually white and glossy, only achieves about 70% reflectivity in the solar spectrum. It reflects a fair quantity of visible light, resulting in its white outward appearance, but its reduced reflectivity to IR solar radiation below 2.5 μm greatly degrades its performance. The TiO_2_ performs similarly to HAP materials across most solar wavelengths, but its normalized reflectivity suffers from a performance drop-off at high UV wavelengths. In contrast, HAP has far greater UV reflectance, allowing both the HAP fibers and HAP/Gamsol paint to outperform the TiO_2_ in terms of reflectivity.

### Cooling Performance

**Figure 6:**
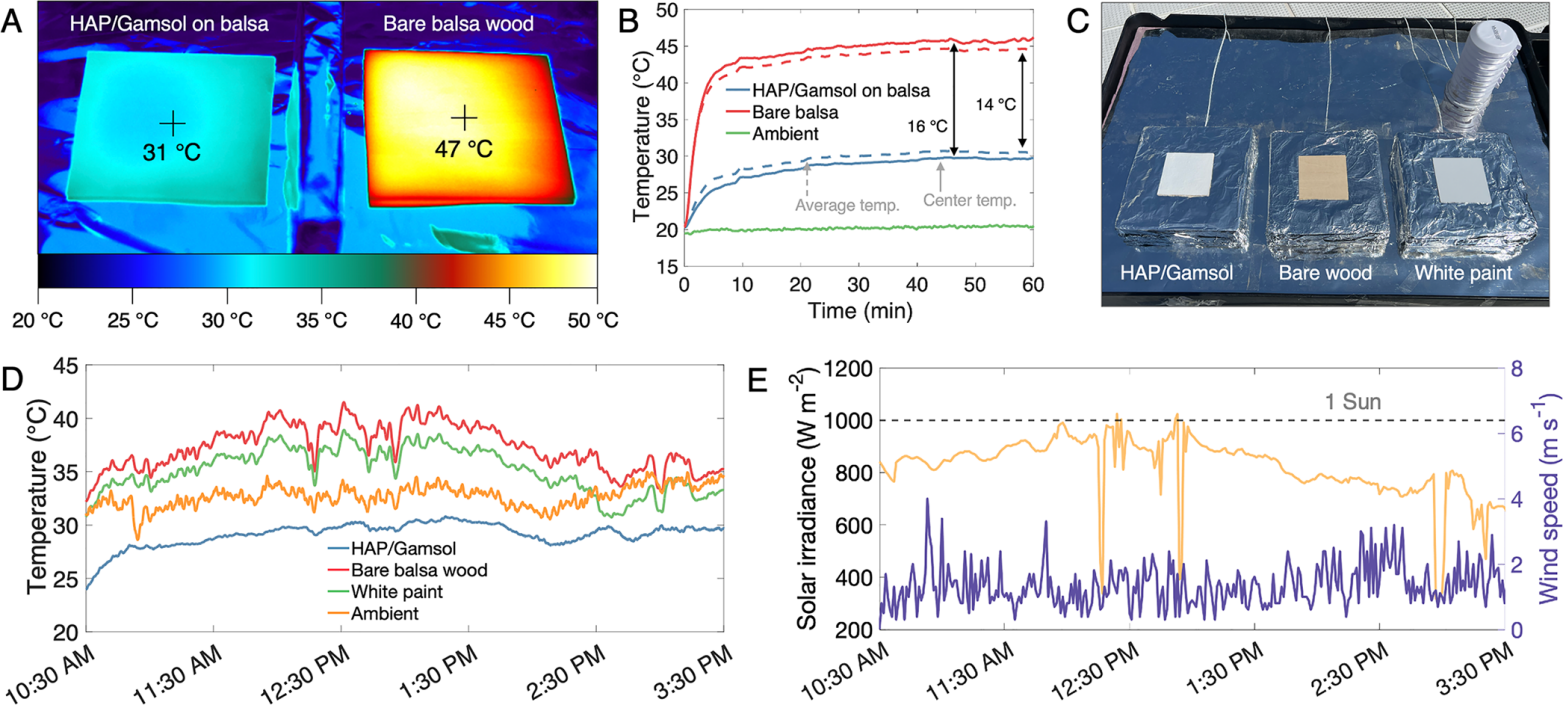
Cooling performance. (A) Infrared camera image of indoor temperature test at 45 min. (B) Thermal response of HAP/Gamsol paint on balsa wood with respect to bare balsa wood during the 1 h indoor test. (C) Experimental setup and comparison samples for the outdoor cooling test. Outdoor testing was done using (D) temperature results and (E) weather conditions.

To demonstrate the cooling abilities of the HAP/Gamsol paint mixture, both indoor and outdoor cooling tests are performed. For the indoor test, two samples are placed under the solar simulator on an insulative polystyrene (PS) sheet (Figure 6a). The sheet is covered by aluminum foil to reduce absorption by the base. The solar simulator is calibrated to 1000 W m^–2^ (1 sun) using a solar irradiance meter. The calibration is performed with the foil placed on the insulation foam to account for the impact of multiply reflected radiation. Samples must be placed fairly close together to receive identical solar irradiation, so an additional PS layer, also covered by foil, is positioned vertically between the two samples. This thermally isolates the two samples to reduce intersample heat transfer. Sample temperatures are monitored by using a thermal camera, while the ambient temperature is measured by using a thermocouple.

For the indoor test, temperatures of the HAP/Gamsol paint on a balsa wood substrate (8 × 8 cm) are compared to an identically sized bare balsa substrate. Both samples reach a steady state after about 40 min. At this point, the HAP/Gamsol paint shows a temperature reduction of about 15 °C compared to the bare balsa substrate. For this test, the cooling performance is owed primarily to the high reflectivity of the HAP/Gamsol paint.

To prove that the HAP/Gamsol mixture functions as an effective passive cooling paint, an outdoor cooling test is performed. Three samples are employed for this comparative test: the HAP/Gamsol on balsa, bare balsa, and commercial white paint on balsa. All samples are placed on polystyrene blocks covered with aluminum foil. Thermocouples measure the backside sample temperatures at the center points of the samples. Due to the thick insulation layer below and relatively low conductive resistance of the samples, the backside temperature provides a very reliable indication of the temperature on the top surfaces of the samples. The test is conducted on June 30, 2023 from 10:30 AM to 3:30 PM, on the rooftop of the Snell Engineering Center (Boston, MA). A weather station is used to monitor weather conditions throughout the test (Figure S3).

During the entirety of the test, the temperature of the HAP/Gamsol painted sample remains below the temperatures of ambient air and the other two samples. All temperatures generally trend with solar intensity, which peaks at intensities just above 1 sun near the middle of the test period. The average subambient temperature drop of the HAP/Gamsol sample is 3.7 °C, and it frequently achieves drops greater than 5 °C during periods of high solar intensity. The cooling power of the HAP/Gamsol paint during the outdoor test is also evaluated by applying eqs 1–5. Measured temperatures and weather conditions are combined with theoretical heat transfer equations, which yield an average cooling power of 75 W m^–2^ during the outdoor test. A peak cooling power close to 100 W m^–2^ is obtained shortly after 1:30 PM, primarily due to a reduction in convective heat transfer at this time. Further details about calculation methods and assumptions are provided in the Supporting Information (Supplementary Note 1 and Figures S4–S6). This experimental demonstration shows clearly how the HAP/Gamsol paint can provide vital cooling for structures throughout the day, reducing both overall A/C use as well as peak A/C demand.

### Recyclability and Durability

To emphasize the benefits of HAP as a passive cooling material, further tests are conducted to evaluate the recyclability, adhesion performance, and water resistance of HAP-based paints. Due to the fire resistance of the HAP nanofibers, the HAP can be recovered from the HAP/Gamsol mixture through a high-temperature baking process. The HAP/Gamsol paint is removed from the substrate using a razor, transferred to an oven, and baked at 900 °C for 4 h. The HAP itself is thermally stable at this temperature, but carbon-based compounds such as oleates, which are present on the surface after synthesis and/or paint bonding/drying will be destroyed.^31^ Thus, the HAP fibers themselves can be reused in paints and other applications.

**Figure 7:**
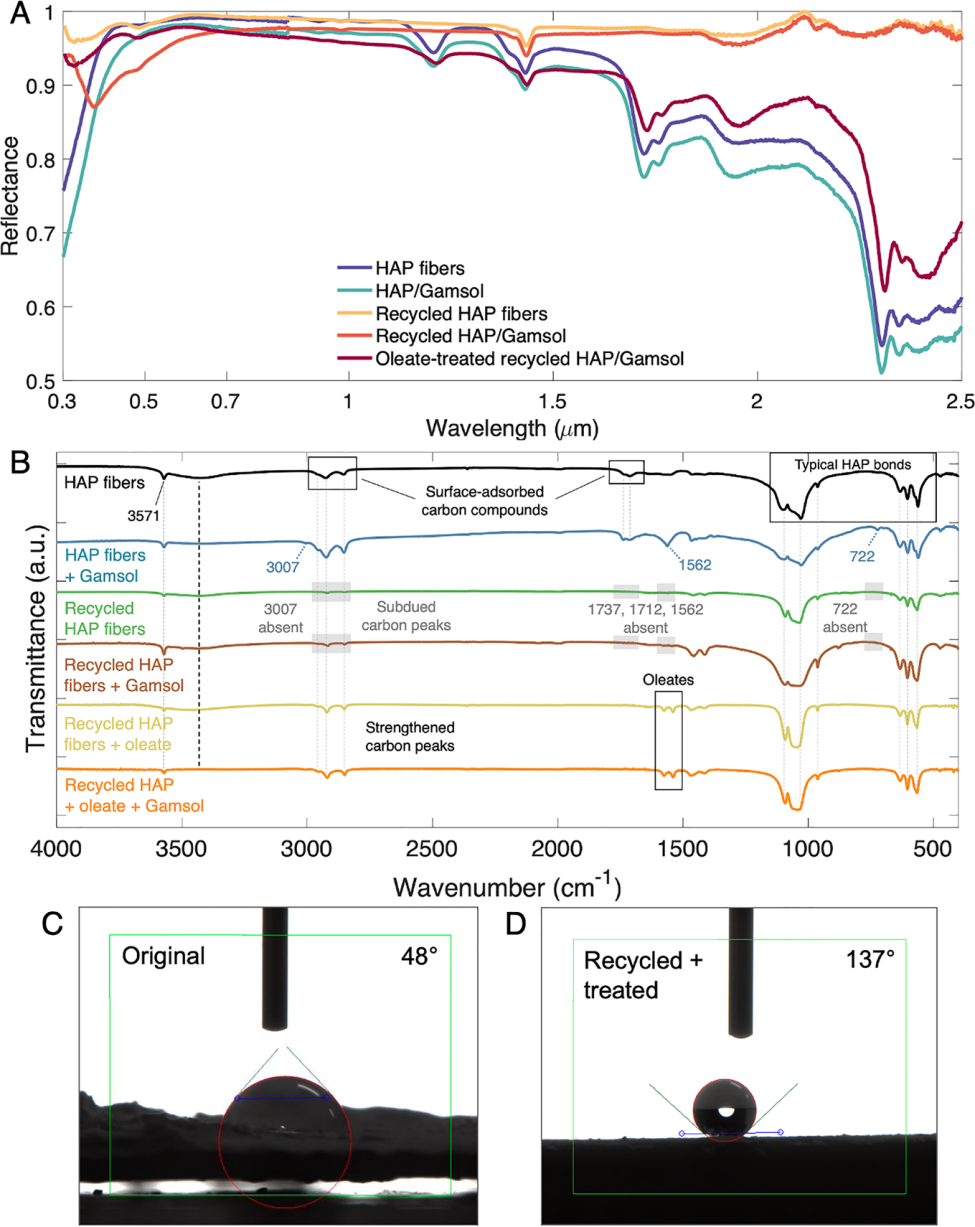
Recyclability of HAP-based paint. (A) Spectral reflectance and (B) FTIR transmittance of HAP samples at different stages in the recycling process. Static contact angles of the (C) original and (D) recycled and oleate-treated HAP/Gamsol paints.

Interestingly, the reflectivity of the HAP fibers is significantly improved after recycling (Figure 7a). This is primarily a factor of surface-adsorbed oleates and oleic acid, which are present after the initial synthesis process. These compounds slightly lower the reflectivity performance of HAP nanofibers, especially in the infrared, compared to recycled HAP nanofibers, which do not contain these surface compounds. However, the unique bonding ability of HAP with Gamsol is eliminated when these surface compounds are absent. Instead of forming a smooth, hardened surface as the original HAP/Gamsol mixture has, the recycled HAP fibers exhibit poor bonding with the Gamsol, as seen in the adhesion test images (Figure S7). This is attributed to the absence of significant surface oleate structures, evidenced by the low intensity of associated FTIR peaks (e.g., 2956, 2925, 2856, 1465, and 722 cm^–1^). The minor difference in the reflectance spectra (Figure 7b) also shows a lack of solar absorption expected from hydrocarbon compounds. Small signal increases at some FTIR peak locations can be attributed to small amounts of the alkane, which may not have evaporated. Also, in unbonded fibers, the broad O–H stretching peak around 3400 cm^–1^ still exists. In the nonrecycled sample, this peak was reduced significantly after successful bonding with Gamsol (likely due to additional oleate adsorption as well as newly emergent intermolecular interactions between surface compounds). However, for recycled HAP, the O–H stretch does not diminish, emphasizing the fact that the proposed bonding mechanism does not take place. Furthermore, the olefinic C–H stretching peak of 3007 cm^–1^ that emerges in all samples after Gamsol or oil drying is notably absent from the recycled HAP/Gamsol mixture. These factors provide a clear indication that the bonding mechanism for HAP/Gamsol hinges on the presence of surface-adsorbed oleates. To further support the suggested oleate bonding phenomenon, two other compounds are combined with Gamsol to attempt to make a reflective coating: TiO_2_ and cellulose (Figure S8). Neither of these substances will have surface-adsorbed oleate compounds, and consequently, neither substance forms a hardened, paint-like surface after combining with Gamsol and drying.

To remedy this bonding issue, oleates can be easily readsorbed onto the surface, due to the presence of calcium ions in the hydroxyapatite.^48,54^ To reuse the HAP fibers as a pigment in the HAP/Gamsol formulation, the recycled HAP is magnetically agitated in a 0.02 mol L^–1^ solution of sodium oleate for 1 h. Thereafter, it is filtered using vacuum filtration and dried at 60 °C for 4 h.^43^ The resulting FTIR transmittance measurements (Figure 7b) illustrate that the adsorption of oleates on the HAP surface is successful. After this treatment, characteristic CH_2_ and CH_3_ peaks from the oleate compounds re-emerge at 2956, 2925, and 2856 cm^–1^, as well as the methylene peaks in the region of 1455–1470 cm^–1^.^47^ The new doublet at 1540 and 1576 cm^–1^ provides the most compelling evidence for oleate adsorption, as this is a well-known characteristic peak of calcium oleate.^47^

To conclude the recyclability study, oleate-treated recycled HAP fibers are redispersed in Gamsol and spread on a substrate for drying, following the original procedure. This yields a stable, hardened surface just as the original HAP/Gamsol paint does (Figure S7). The reflectivity of the recycled paint is very similar to that of the initial paint sample, albeit with a slightly modified performance in the UV and IR regions. This is likely based on differences in the morphologies and adsorption environments of the oleate layers, which are well-known to impact surface structure characteristics.^46−48^ Even so, the normalized reflectivity remains unchanged at 95% for the recycled oleate-deposited HAP/Gamsol paint.

This data clearly support the necessity of oleate surface structures in the formation of a mechanically stable HAP/Gamsol paint. However, the FTIR transmittance measurements for the recycled and treated samples show evidence of bonding through a slightly modified mechanism as compared to the original samples. The only notable change after Gamsol addition to the recycled sample is the reduction in water content. There is no evidence of further oleate adsorption onto the surface after the Gamsol is added; all other characteristic FTIR peaks are effectively unchanged. Furthermore, the hydrophobicity of this sample contrasts with the original HAP/Gamsol sample (Figure 7C, D). These factors indicate that the recycled oleate treatment forms a well-ordered oleate surface structure with the nonpolar chains of the oleate oriented away from the surface.^54,63^ Thus, the addition of Gamsol appears to also facilitate oleate chain interaction for existing surface-adsorbed oleates and not just newly created ones. The hydrophilic alkanes may act as a nonpolar bridge between oleate chains, which is maintained after Gamsol evaporation.

Finally, to provide further support for the proposed bonding mechanisms of initial and recycled HAP/Gamsol paints, pure sodium oleate (NaOl) is studied. NaOl is crushed using a mortar and pestle before combining with Gamsol, and spreading on a substrate following the original procedure. Once again, the resulting composite forms a hard, stable surface after drying, evidencing that the oleates themselves provide the major structural support for the HAP/Gamsol paint. FTIR transmittance spectra are measured for pure NaOl, NaOl immediately after mixing with Gamsol (before drying), and the NaOl/Gamsol mixture (Figure S9). The anticipated characteristic peaks (e.g., 2956, 2925, 2856, 1562, 1465, and 722 cm^–1^) are obvious in all samples. The spectra of pure NaOl and the NaOl/Gamsol mixture after drying are nearly identical, illustrating that no chemical reactions occur during bonding with Gamsol. However, behaviors of the O–H stretch during bonding become obvious through this test. When the Gamsol is added to the mixture (prior to drying), it appears to displace water significantly based on the absent O–H stretch resonance, supporting the proposed bonding mechanisms outlined previously. Furthermore, this O–H stretch returns to its original magnitude after drying, showing that the oleates may absorb some atmospheric water. In contrast, the various HAP/Gamsol mixtures show reduced water content after bonding with Gamsol. Thus, the reduced water content after bonding is clearly due to a suppression of HAP water absorbance, as opposed to that of the surface oleates. Therefore, we posit that both additional surface-adsorbed oleates and additional nonpolar interfiber oleate interactions suppress the ability of HAP to absorb water, due to added surface coverage and hydrophobicity.

The results of adhesion and water resistance tests can be used to evaluate the use of HAP-based paints in applied scenarios and compare them with commercial paints. For the adhesion test, the practices of ASTM D3359 are used to compare the ability of the paints to form a mechanically stable structure and bond to the substrate, as described in the Experimental Section. As shown in Figure S7, the commercial paint performs the best of all samples due to its hardened acrylic structure. Barely any residue is visible on the adhesive, besides a faint impression of the cut. In comparison, the HAP/Gamsol paint performs slightly worse but still demonstrates stable bonding within the coating and to the substrate. Small amounts of residue are visible in the cut area and sporadically around the cut. However, this residue is present in very limited amounts, showing that even weak oleate–oleate interactions are capable of maintaining a stable coating structure. After the HAP/Gamsol coating is recycled via a high-temperature heating process, it does not form a coating when combined with Gamsol, as previously noted. There is no significant force holding the fibers together, resulting in the adhesive being completely covered by the recycled HAP fibers after it is peeled off. When surface-adsorbed oleates are reintroduced onto the fibers, however, the Gamsol is again able to bond the fibers together once again. The addition of surface-adsorbed oleates facilitates a much more stable coating structure when Gamsol is added, as seen for the recycled and oleate treated HAP/Gamsol mixture in Figure S7. While slightly more residue is seen on the recycled HAP/Gamsol paint as compared to the original one, this is attributed to the fact that the original paint possesses a relatively thicker oleate surface layer, indicated by its FTIR signals and hydrophilicity.^48^

Finally, the ability of the HAP/Gamsol paints to resist water is evaluated using a 30 min simulated rain test, where a syringe drips water onto the samples at a constant rate of 70 mL h^–1^ (Figures S10–S12 and Videos S1–S3). Images of selected samples are shown before and after this test in Figure S11. Due its low contact angle, the original HAP/Gamsol paint is largely compromised by prolonged exposure to water, whereby the weak molecular interactions holding the coating together are disrupted by the presence of water on the surface. However, the recycled HAP/Gamsol coating, with its high contact angle introduced by well-ordered oleate surface structures, is able to repel incoming water. This coating experiences no visible degradation after the water test, similar to the commercial-quality acrylic-based paint used for comparison. Its normalized solar reflectance value is also identical before and after the water test (Figure S12). To further exemplify the water resistance of the recycled HAP/Gamsol paint, the sample is subjected to an even heavier flow of 1 L h^–1^ for 30 min. As seen in Figure S13 and Video S4, the hydrophobicity allows this paint to survive with negligible degradation, even after intense water exposure. Thus, while the water resistance ability of the original HAP/Gamsol paint is poor, the surface modification of the HAP fibers which introduces hydrophobicity provides the capability to withstand the impacts of water in an external environment.

## Discussion

Surface-adsorbed oleate compounds appear to have a slightly detrimental impact on near-IR reflectivity in the 1.5–2.5 μm region. However, these compounds also provide a mechanism for the HAP to bond strongly with oil-based paint binders, providing a more practical delivery method for the desirable passive cooling properties of HAP. With proper surface treatment methods, these oleates can also provide critical multifunctionalities for passive cooling applications–namely hydrophobicity, which is highly desirable in such applications as it makes external applications more feasible.^21^ In addition, these carbon-based surface compounds can easily be modified after removal via a high-temperature treatment, expanding applications for recycled HAP. Though paints are generally not recycled and HAP is generally cheap, we note that the exemplification of recycling opportunities can promote future sustainability prospects through potential circular economic practices.

The typical paint binders used in this work, safflower and linseed oils, are generally more reliable for bonding pigments. The peroxidation of their fatty acids provides a strong surface for a much wider range of pigments and does not rely on specific surface structures such as oleates as the Gamsol does. However, the solar reflectivity is reduced greatly for these binders, limiting their use for passive cooling applications. This is obvious in cases such as the linseed oil, which yellows significantly after drying and barely achieves 50% solar reflectance. Even for oil paint binders which exhibit less yellowing due to a lower linoleic acid content,^57^ such as the safflower oil utilized in this work, the overall solar reflectivity is still poor. In contrast, while the ability of Gamsol to act as a binder is limited by the surface characteristics of pigment materials, its ability to facilitate bonding with very little optical performance reduction is extremely valuable.

Furthermore, the flexibility of surface-adsorbed oleates on apatites is shown clearly through this work. In the initial bonding mechanism, a thicker, more amorphous oleate surface layer dominates the paint structure. It provides mechanical strength, but the disordered nonpolar chains and excess molecules within this layer render the surface hydrophilic, which is detrimental for external applications where water resistance is required. For the recycled bonding mechanism, a thinner and far more ordered surface structure results, with properties suggesting near-monolayer oleate coverage.^43,46,48,54^ This provides strong hydrophobicity as well as interfiber bonding via nonpolar oleate chain interactions, both of which provide significantly improved resistance to water. Besides forming a unified surface after Gamsol addition, the fact that the HAP surface can be tuned via a simple surface treatment provides further flexibility in the realm of large-scale paint synthesis. With proper pretreatment, the HAP can be expected to combine well with a variety of other paint media, as it may be modified to interact with both polar and nonpolar media.

Finally, it is worth mentioning the possibility that alkane chains from Gamsol may remain in the structure bonded to the nonpolar hydrocarbon chains of the surface oleates. Based on the lack of significant differences between FTIR spectra before and after Gamsol addition for recycled and oleate-treated HAP fibers, as well as pure NaOl, we do not expect alkanes that contribute significantly to the dried structure to remain after drying. It is difficult to conclude with certainty, as oleates share IR vibrations with alkanes (CH_2_ and CH_3_). However, if a significant quantity of these alkanes did remain, then IR absorption by oleate-unique vibrations (olefinic C = H and carboxyl) would be expected to decrease with respect to those of CH_2_ and CH_3_, which are shared by both compounds. This phenomena is not possible to assess in the initial sample, as the quantity of oleates grows significantly after the addition of Gamsol. However, the transmittance spectra of the recycled and oleate-treated HAP fibers as well as pure NaOl can be used for this purpose (Figures S14 and S15). Indeed, the intensity of peaks shared by alkanes and oleates does not increase with respect to oleate-unique peaks. There exists no significant correlation between the intensity changes of these two peak types before and after Gamsol is added. For this reason, alkanes are not assumed to remain in the paint in any significant quantity after the paint dries.

This work illustrates that oleate-based bonding interactions, although weaker mechanically than the organic cross-linking of typical paint binders, allow for the formation of coatings with minimal optical absorption losses in the solar wavelength region compared to polymeric binders. Such an approach presents great value for practical passive radiative cooling materials, which constantly strive for near-perfect solar reflectance but are often hindered by complex application methods. The bonding behaviors and autoxidation of oleate/alkane mixtures are complex, especially with multiple available avenues for alkane autoxidation, and they are not further investigated in the scope of this work. However, the authors emphasize that this concept presents great opportunity for biological applications, such as drug delivery and implants, as well as for investigating the oxidation and processing of hydrocarbon fuels, especially at room temperature.^66−68^

## Conclusion

In this work, an effective strategy is proposed to transform nanofibrous hydroxyapatite, a passive radiative cooling biomaterial pigment, into an easy-to-apply paint. Tunable oleate surface structures on the hydroxyapatite provide reliable bonding using the alkane compound Gamsol, which is not possible for many other pigments. This unique bonding strategy severely reduces optical losses after paint drying, enabling high-performance passive radiative cooling for a scalable paint-like material. By leveraging this technique, solar reflectance and infrared emittance of 95% and 92%, respectively, are achieved. Both indoor and outdoor tests under solar irradiance are performed to validate the passive cooling performance of the hydroxyapatite/Gamsol paint, illustrating an average subambient cooling performance of 3.7 °C. The pigment can also be recycled and recovered for reuse as a passive cooling pigment or other applications, due to its high-temperature stability. Hydrophobicity can also be implemented easily through recycling and surface treatment processes prior to paint deposition. In summary, this nanofibrous biomaterial-based paint provides a perfect avenue for reducing A/C-related emissions and heat-related illness throughout the world. As efforts accelerate to combat climate change, hydroxyapatite-based materials offer many valuable sustainable benefits, including passive cooling, ecofriendliness, and recyclability, all of which will contribute to a greener future through carbon emission reduction, circular economic practices, and more equitable access to cooling technology.

## Experimental Methods

### Materials

Calcium chloride (CaCl_2_), oleic acid (C_18_H_34_O_2_, natural, FCC), sodium dihydrogen phosphate (NaH_2_PO_4_ · 2H_2_O, anhydrous, ≥ 97%), sodium oleate (NaOl), and sodium hydroxide (NaOH) are obtained from Sigma-Aldrich. Gamsol, safflower oil, and linseed oil are obtained from Gamblin. Titanium dioxide nanopowder (TiO_2_, anatase, 99.9%, 100 nm) is obtained from US Research Nanomaterials (US3411). Rust-Oleum white and balsa wood are obtained from Amazon. Ethanol is obtained from Lab Alley.

### Synthesis of HAP Fibers

Hydroxyapatite nanofibers are fabricated using a typical solvothermal synthesis method (Figure 1b)^31^ using a ZZKD FCF-2L Stainless Steel Autoclave Reactor equipped with a PTFE liner. 172.8 g of oleic acid is combined with 172.8 g of ethanol in a beaker. This mixture is stirred using magnetic stirring for 20 min at 500 rpm. Next, three other solutions are prepared. The first solution is made by combining 3.17 g of CaCl_2_ and 240 mL of DI water. The second solution is made by combining 14.4 g of NaOH and 240 mL of DI water. The third solution is made by combining 3.36 g of NaH_2_PO_4_ · 2H_2_O with 120 mL of DI water. All three of these solutions are separately stirred using magnetic stirring at 500 rpm for 10 min. Subsequently, these three solutions are slowly poured into the oleic acid/ethanol mixture in sequence, followed by continued magnetic stirring of the combined solution for another 10 min. These steps produce the sodium oleate precursor mixture.

Next, the sodium oleate precursor is poured into a Teflon-lined 2 L autoclave reactor. The reactor is sealed and heated to 180 °C for 4 h. After heating, the sealed reactor is cooled to 60 °C, after which the mixture is transferred from the reactor to a glass beaker, along with 1 L of ethanol. This mixture is kept at 60 °C for 2 h under weak magnetic stirring (100 rpm) for precipitation. During this time, the less-dense HAP nanofibers begin to rise to the top of the solution, forming a white, cloud-like mass. Finally, the entire mixture is filtered using vacuum filtration through a 2 μm filter paper. Rinsing with DI water and/or ethanol is omitted to facilitate paint drying and hardening, as will be explained later in the text. After being heated overnight at 60 °C to evaporate any remaining ethanol and water, the solid HAP nanofibers are obtained in a sheet-like form. Approximately 3.8 g of HAP fibers is produced using the aforementioned procedure.

### Fabrication of HAP Fiber-Based Paints

To yield the HAP-based paints, HAP nanofibers are ground using a mortar and pestle until they resemble a powder (SEM images) to help disperse them within the chosen matrix materials. Using a painting trowel on a plastic mixing tray, the ground HAP fibers are combined with several drops of various types of oil (e.g., Gamsol, linseed oil, and safflower oil). The ground fibers are mixed into the oil using a trowel until a moldable paste is produced. If the mixture is too dry to spread on the desired medium, additional oil is added; similarly, if the mixture becomes too wet, additional ground fibers are added. Once mixed to the targeted consistency, the mixture is spread on the substrate with a thickness of 0.5 mm and left to dry for 5 days.

### Characterization Procedures

Reflectance spectra of samples from 300–2500 nm are obtained using the Jasco V770 spectrometer, equipped with the Jasco ISN-923 integrating sphere at an angle of 6°. Reflectance spectra in the mid-IR (2.5–20 μm) are obtained using the Jasco FTIR 6600. Based upon previous investigation into the spectral properties of the HAP nanofibers and the thickness of the paint layer, the paint layer can be assumed to be opaque (transmittance equal to zero). The FTIR transmissivities were also obtained using a Jasco FTIR 6600 instrument by embedding a small amount of sample within a potassium bromide window. Thermal conductivities are measured using a HotDisk TPS 2500s. The contact angle measurement is performed using the SINDIN SDC-350 contact angle meter. SEM images are obtained using the Supra 25 SEM with an acceleration voltage of 5 kV. Chemical surface characterizations are performed using the Bruker Quantax EDS with an acceleration voltage of 20 kV. A 10 nm layer of gold/palladium is deposited on the samples prior to imaging with SEM and EDS characterization. Temperature data and thermal camera images are taken using the FLIR A655C thermal camera with a resolution of 640 × 480 using a 25° lens. Additional temperature data are also obtained using K-type thermocouples. A Newport 94801a solar simulator is used for indoor cooling tests, which is calibrated using a TES 132 Solar Power Meter prior to each test. For the adhesion tests, the procedures of ASTM D3359 are adopted. Coatings on wood substrates are cut with an “X” shape, and clear adhesive tape is pressed evenly onto the surface using a 50 g mass. The tape is then peeled off, and the area of the coating residue on the tape is evaluated. For the water resistance tests, the backside of samples is adhered to an acrylic plate at a 30° angle to mimic the slope of a typical roof. Using a syringe pump, DI water is dripped onto each sample at a rate of 70 mL h^–1^ from a height of approximately 100 mm. The intensified water test performed on the recycled HAP/Gamsol sample is run in similar conditions with a flow rate of 1 L h^–1^. Each test is run for 30 min, and the sample surfaces are compared before and after the test to evaluate their resistance to water damage. The water resistance of samples is evaluated directly after the adhesion test to introduce some wear into the coatings as they may experience in external environments over time.

## Supporting Information Available

Images of experimental setups, a description of the calculation of cooling power during the outdoor test, heat transfer component results during the outdoor test, supplementary FTIR data, results of adhesion and water resistance tests, and dynamic contact angle results (PDF)
Video S1 contains footage of the commercial paint during the water resistance test (MOV)
Video S2 contains footage of the HAP/Gamsol paint during the water resistance test (MOV)
Video S3 contains footage of the recycled HAP/Gamsol paint during the water resistance test (MOV)
Video S4 contains footage of the recycled HAP/Gamsol paint during the intensified water resistance test (MOV)

## Supplementary Material

am4c01383_si_001.pdf

am4c01383_si_002.mov

am4c01383_si_003.mov

am4c01383_si_004.mov

am4c01383_si_005.mov
