# Supplementary material for: Nanofibrous Biomaterial-Based Passive Cooling Paint Structurally Linked by Alkane-Oleate Interactions
Source: ACS Appl Mater Interfaces. 2024 Mar 1;16(10):12717–30. doi: 10.1021/acsami.4c01383 (PMC10941070; doi:10.1021/acsami.4c01383)
Supplement: Supplementary file 1 — am4c01383_si_001.pdf [file am4c01383_si_001.pdf]

Supporting information: Nanofibrous biomaterial-based passive cooling paint structurally linked by alkane-oleate interactions

Andrew Caratenuto[1], Kyle Leach[1], Yang Liu[1], Yi Zheng[1,2*]

[1]Department of Mechanical and Industrial Engineering, Northeastern University, Boston, Massachusetts 02115, USA.
[2]Department of Chemical Engineering, Northeastern University, Boston, Massachusetts 02115, USA.

*Corresponding author: y.zheng@northeastern.edu

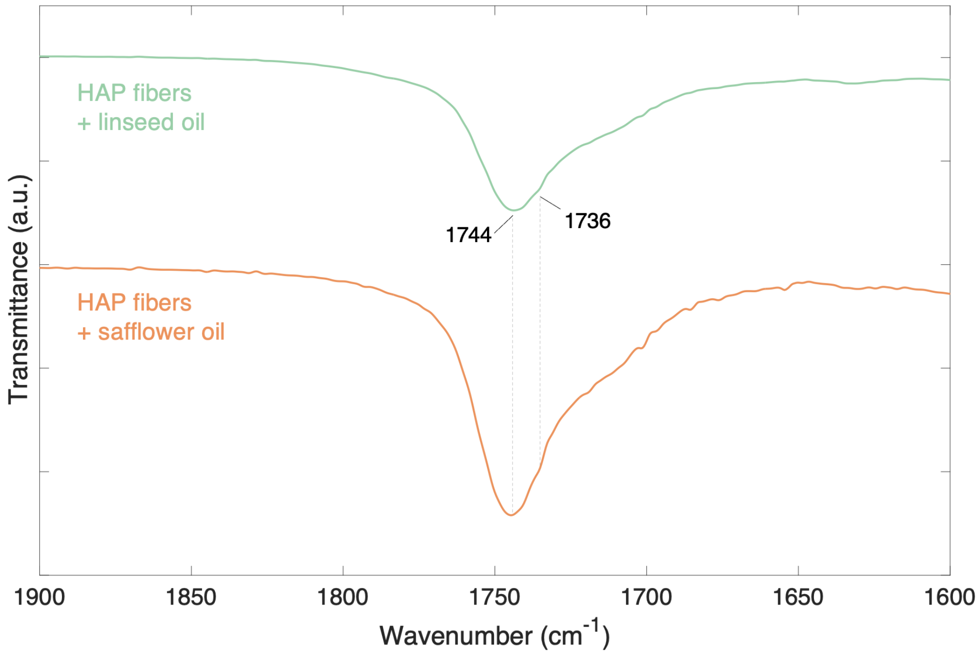

Figure S1 Close-up of linseed oil and safflower oil FTIR transmittance measurements.

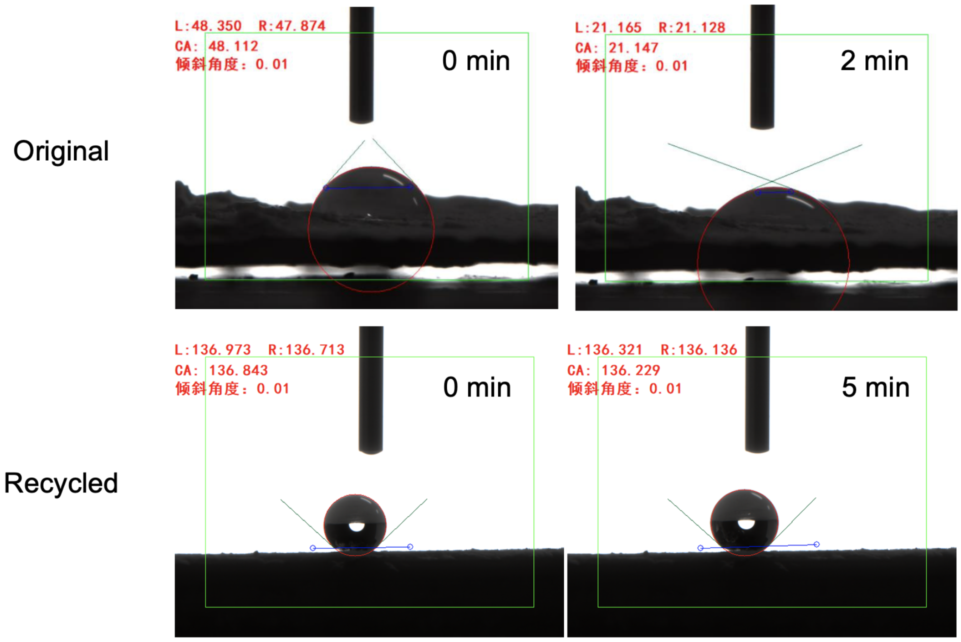

Figure S2 Dynamic contact angles of initial and recycled HAP/Gamsol paints.

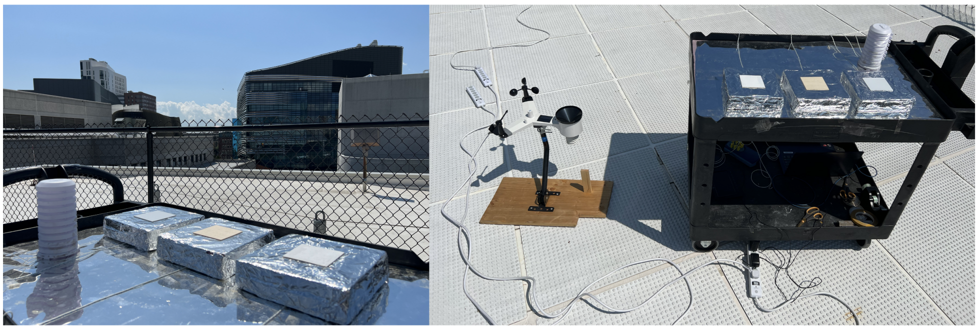

Figure S3 Images of outdoor cooling experiment setup.

Supplementary Note 1

Calculation of cooling power

Net cooling power is calculated from the following equations, described in the main text:

$$q''_{net} = q''_{rad,out}(T_s) - q''_{rad,in}(T_\infty) - q''_{conv}(T_s, T_\infty) - q''_{cond}(T_s) - q''_{solar} \quad (1)$$

$$q''_{rad,out}(T_s) = 2\pi \int_0^{\pi/2} \int_0^{\infty} I_{BB}(T_s, \lambda)\varepsilon_s(\lambda, \theta) sin(\theta) cos(\theta) d\theta d\lambda \quad (2)$$

$$q''_{rad,in}(T_\infty) = 2\pi \int_0^{\pi/2} \int_0^{\infty} I_{BB}(T_\infty, \lambda)\varepsilon_s(\lambda, \theta)\varepsilon_\infty(\lambda, \theta) sin(\theta) cos(\theta) d\theta d\lambda \quad (3)$$

$$q''_{solar} = \int_0^{\infty} I_{solar}(\lambda)\varepsilon_s(\lambda, \theta_{solar})\, d\lambda \quad (4)$$

$$I_{BB} = \frac{2hc^2}{\lambda^5(e^{\frac{hc}{\lambda kT}} - 1)}, \quad (5)$$

Convective heat transfer is given by

$$q''_{conv} = h(T_s - T_\infty), \quad (6)$$

where h is the convective heat transfer coefficient. This coefficient is obtained for each data point by evaluating the Nusselt number. By assuming a flat plate under external flow with uniform surface heat flux, the correlation

$$\overline{Nu}_L = 0.680 Re_L^{1/2} Pr^{1/3}, \quad (7)$$

is used, where Re_L and Pr are the Reynolds and Prandtl numbers, respectively[1]. Nearly all wind speed data points during the outdoor test are above 0.5 m/s, and the average wind speed is about 1.4 m/s, so it is not necessary to consider natural convection.

Conduction is considered negligible in this calculation for several reasons. The low thickness and relatively high thermal conductivity of the HAP/Gamsol sample provide a very low thermal resistance between the top and bottom surfaces. The contact area between the bottom surface of the sample and the insulation foam below is also very minimized, limited to a small amount of double-sided adhesive around the bottom border of the sample. Also, the insulation foam and aluminum foil upon which the samples are mounted help minimize temperature difference between the bottom surface of the sample and the mounting surface.

Figs. S4 – S6 show the results of these calculations over the outdoor test period. Results are averaged over periods of 15 min to establish a clearer representation of cooling power and other heat transfer phenomena during the test. Several phenomena provide support for the accuracy of this calculation. Radiation heat transfer components vary only slightly over the test period, as the radiative output is dependent only

on sample or ambient temperatures. The convective heat transfer is inversely proportional to the temperature difference between the sample and the ambient, as expected. The values for the convection coefficient are also in a reasonable range for forced air convection.

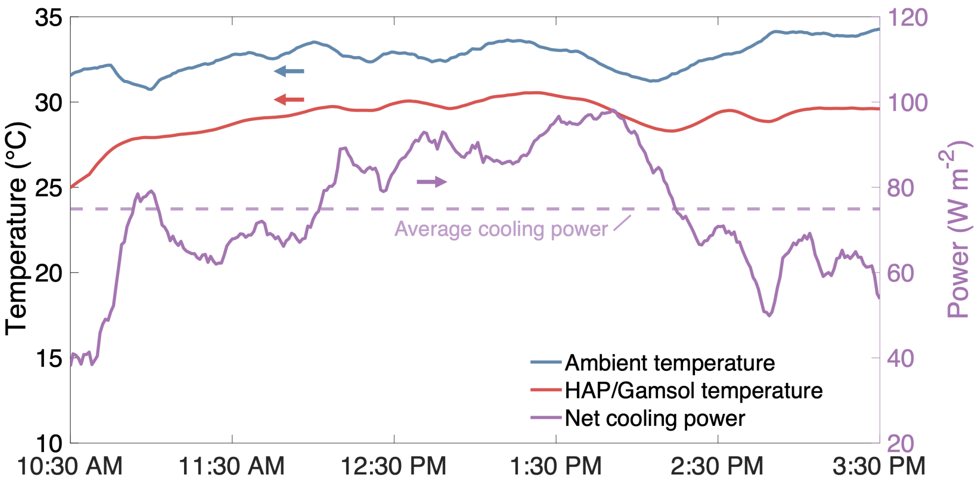

Figure S4 Temperatures and theoretically calculated cooling power during outdoor test.

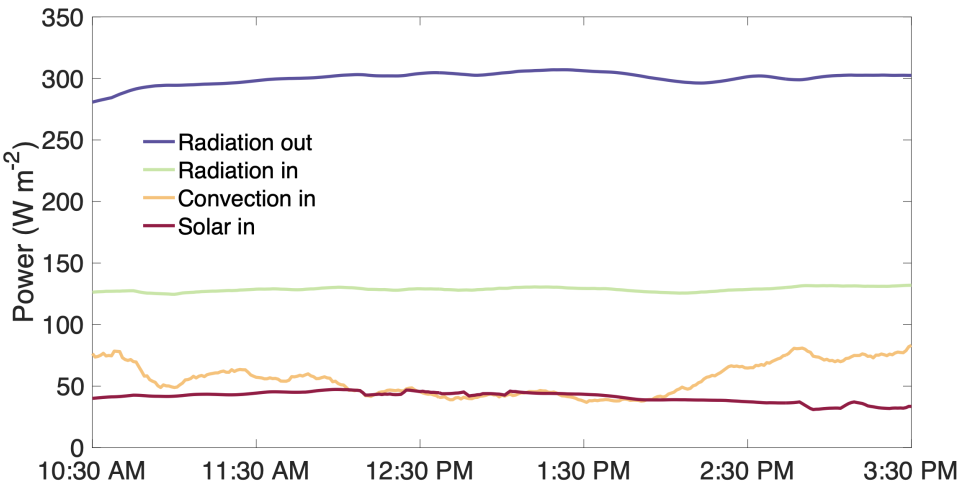

Figure S5 Heat transfer components used for calculation of net cooling power.

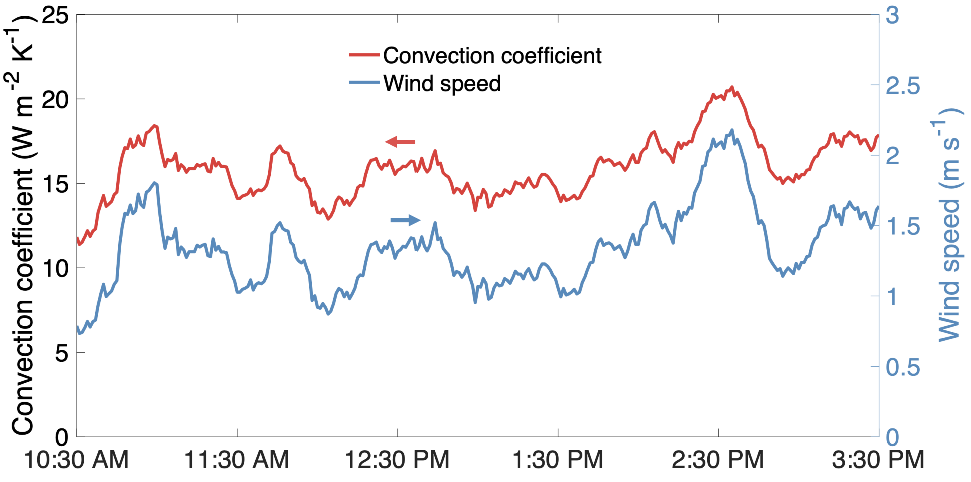

Figure S6 Convection parameters for cooling power calculation.

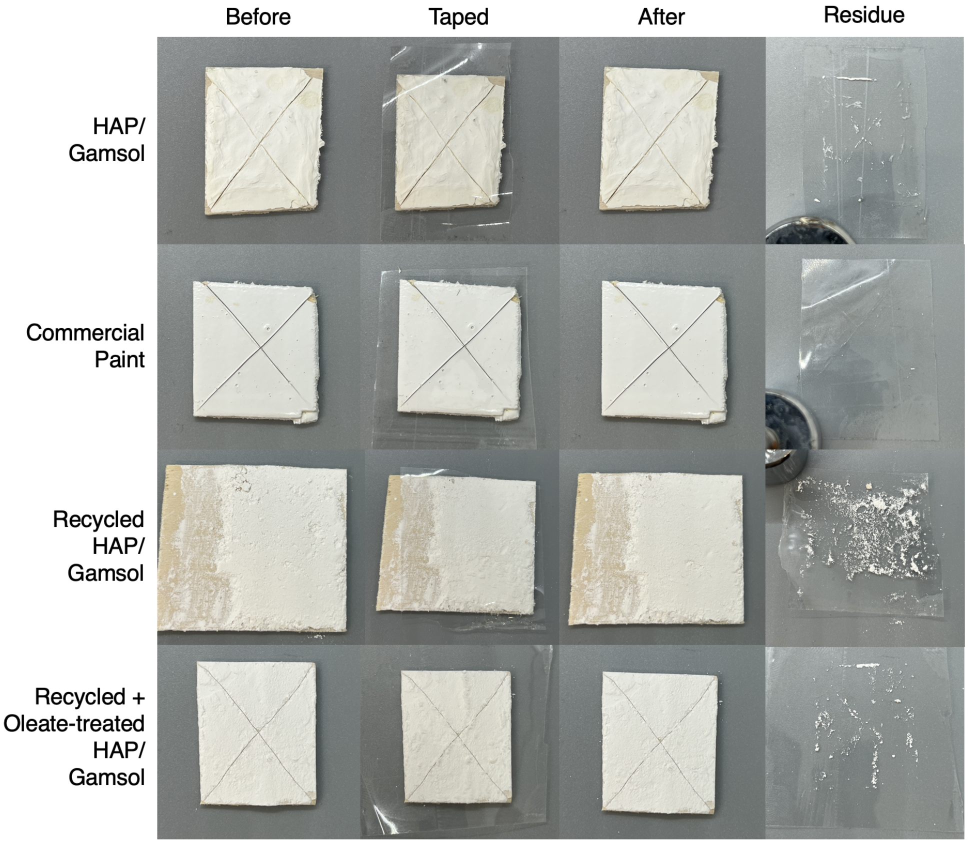

Figure S7 Results of adhesion test.

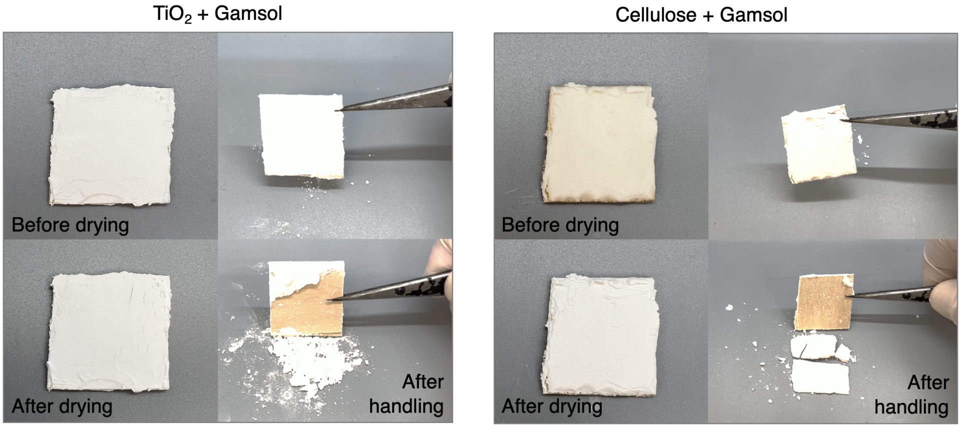

Figure S8 Dried TiO_2 and cellulose-based mixtures after combination with Gamsol.

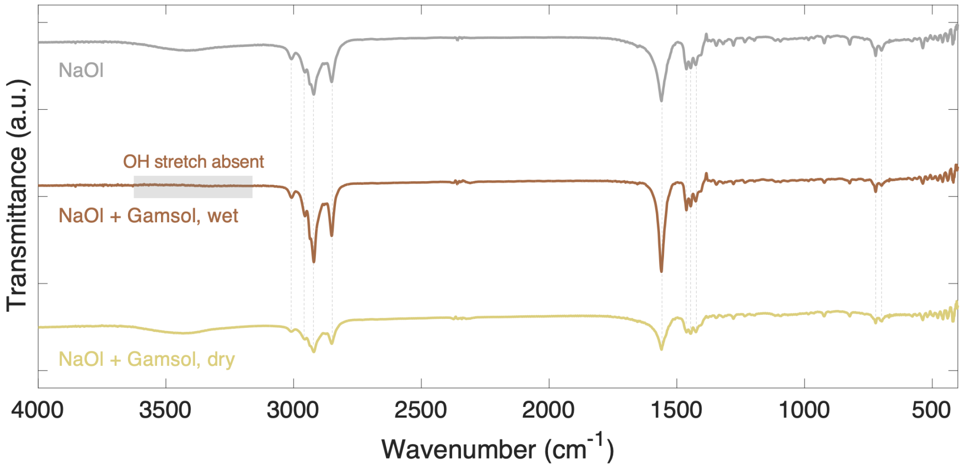

Figure S9 FTIR transmittance spectra of sodium oleate (NaOl), NaOl + Gamsol (wet), and NaOl + Gamsol (dry).

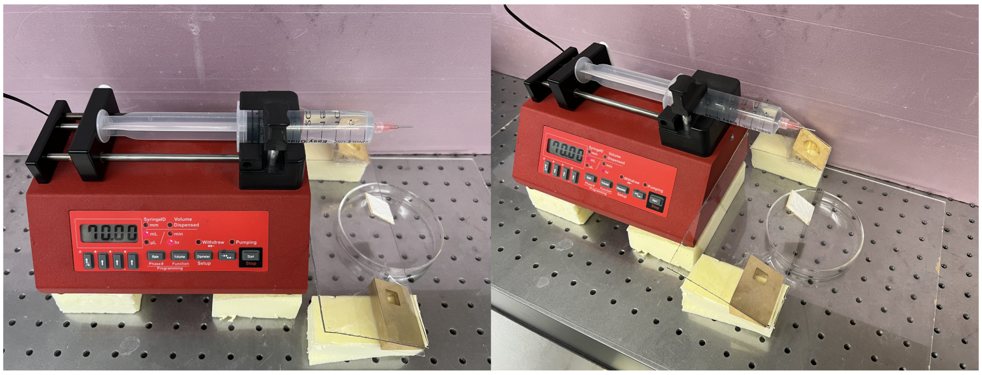

Figure S10 Water resistance test setup, designed to simulate outdoor exposure to rain.

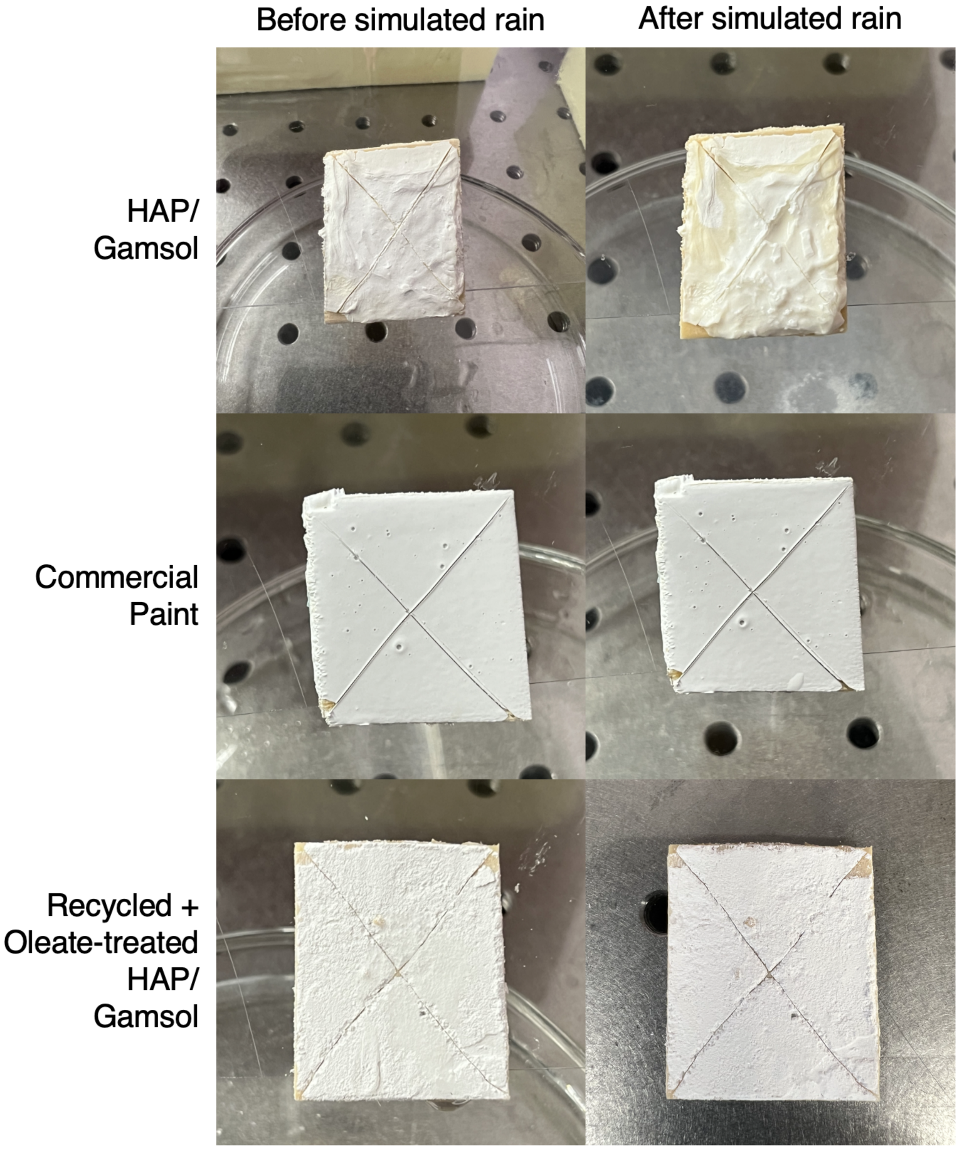

Figure S11 Images of selected samples before and after the water resistance test.

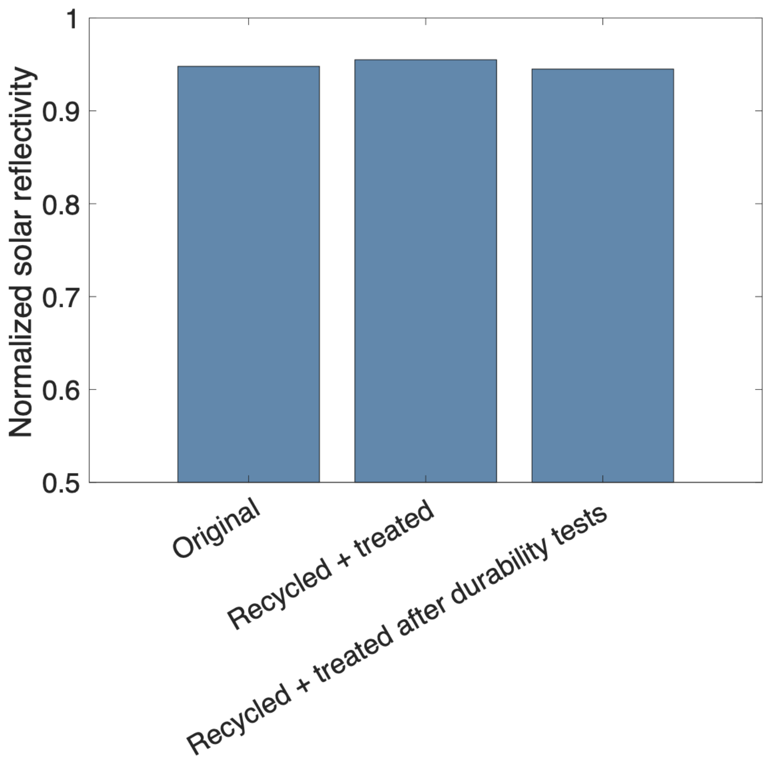

Figure S12 Normalized reflectivity of original HAP/Gamsol paint and recycled and treated HAP/Gamsol paint before and after water resistance test. The original HAP/Gamsol paint was not tested after the water resistance test as it was partially compromised.

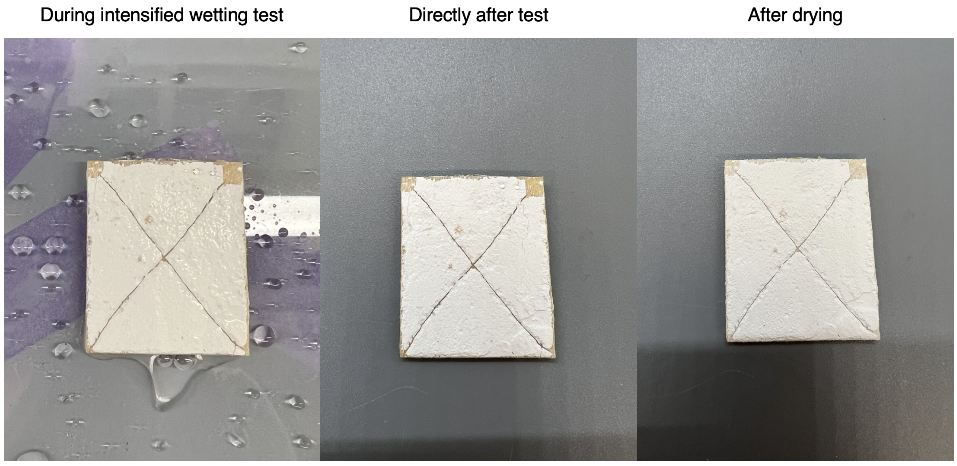

Figure S13 Recycled and oleate-treated HAP/Gamsol paint during the intensified water resistance test, directly after the test, and after drying, showing no degradation.

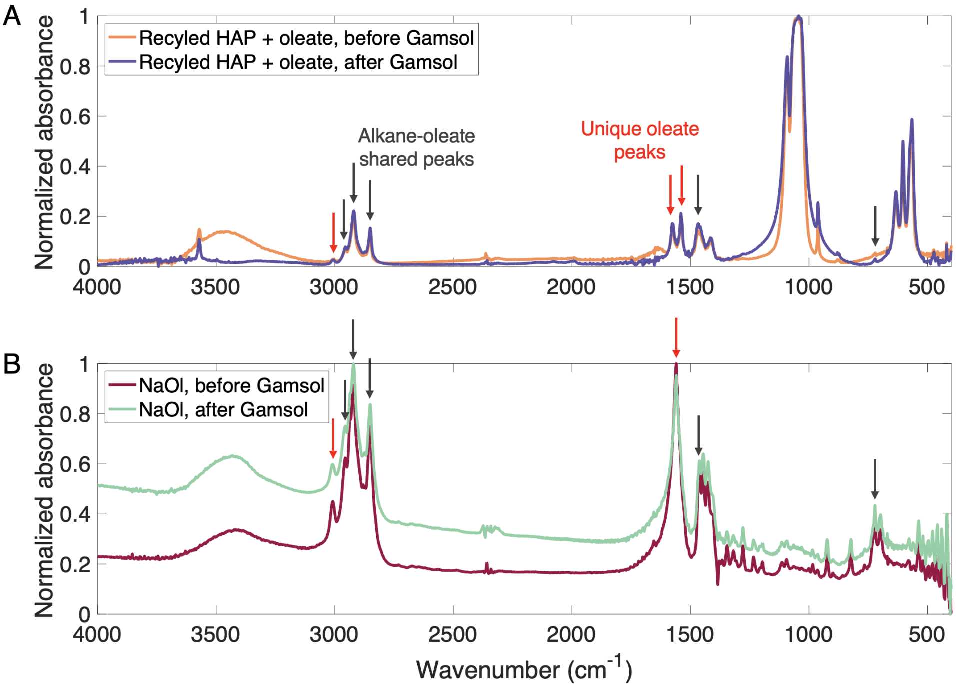

Figure S14 FTIR transmittance spectra of (A) recycled HAP after oleate treatment and (B) NaOl, before and after combining with Gamsol and drying. Gray arrows indicate CH_2 and CH_3 peaks shared by oleates and alkanes, while red arrows indicate carboxyl and olefinic C=H peaks unique only to oleates.

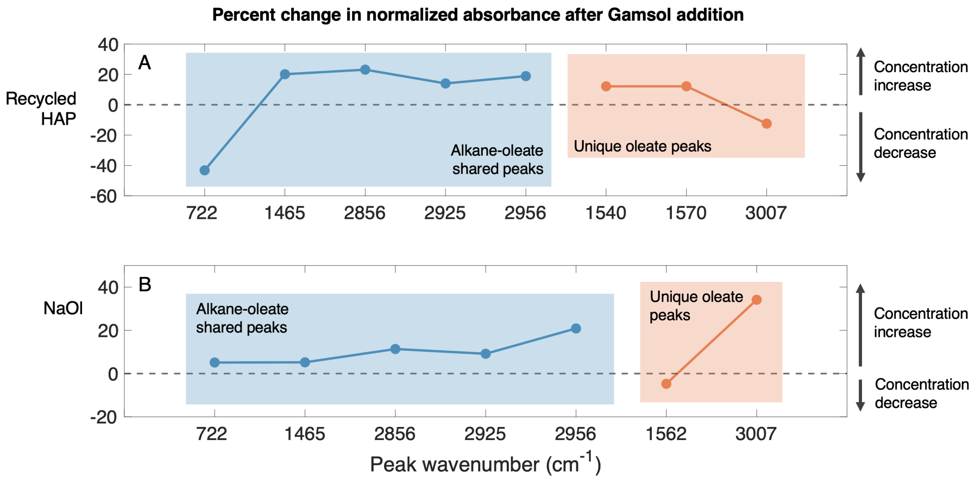

Figure S15 Percent change in normalized absorbance peak intensity after Gamsol addition and drying, for (A) recycled HAP after oleate treatment and (B) NaOl. A concentration increase can be inferred from positive values, and vice versa for negative values. While most alkane-oleate shared peaks rise slightly after Gamsol is added, oleate-unique peaks both increase and decrease. Thus, concentration differences are not significant, and alkanes are likely not actively participating in the dried structure.

References

[1] Incropera FP, DeWitt DP, Bergman TL, et al (1994) Fundamentals of Heat and Mass Transfer. John Wiley & Sons, Hoboken, NJ
